# Quinuclidine-Based Carbamates as Potential CNS Active Compounds

**DOI:** 10.3390/pharmaceutics13030420

**Published:** 2021-03-20

**Authors:** Ana Matošević, Andreja Radman Kastelic, Ana Mikelić, Antonio Zandona, Maja Katalinić, Ines Primožič, Anita Bosak, Tomica Hrenar

**Affiliations:** 1 Institute for Medical Research and Occupational Health, Ksaverska Cesta 2, HR-10 000 Zagreb, Croatia; amatosevic@imi.hr (A.M.); azandona@imi.hr (A.Z.); mkatalinic@imi.hr (M.K.); 2 Department of Chemistry, Faculty of Science, University of Zagreb, Horvatovac 102A, HR-10 000 Zagreb, Croatia; andreja.radman@gmail.com (A.R.K.); ana.mikelic@chem.pmf.hr (A.M.); ines.primozic@chem.pmf.hr (I.P.)

**Keywords:** Alzheimer’s disease, acetylcholinesterase, butyrylcholinesterase, inhibition, covalent binding, cytotoxicity

## Abstract

The treatment of central nervous system (CNS) diseases related to the decrease of neurotransmitter acetylcholine in neurons is based on compounds that prevent or disrupt the action of acetylcholinesterase and butyrylcholinesterase. A series of thirteen quinuclidine carbamates were designed using quinuclidine as the structural base and a carbamate group to ensure the covalent binding to the cholinesterase, which were synthesized and tested as potential human acetylcholinesterase (AChE) and butyrylcholinesterase (BChE) inhibitors. The synthesized compounds differed in the substituents on the amino and carbamoyl parts of the molecule. All of the prepared carbamates displayed a time-dependent inhibition with overall inhibition rate constants in the 10^3^ M^−1^ min^−1^ range. None of the compounds showed pronounced selectivity for any of the cholinesterases. The in silico determined ability of compounds to cross the blood–brain barrier (BBB) revealed that six compounds should be able to pass the BBB by passive transport. In addition, the compounds did not show toxicity toward cells that represented the main models of individual organs. By machine learning, the most optimal regression models for the prediction of bioactivity were established and validated. Models for AChE and BChE described 89 and 90% of the total variations among the data, respectively. These models facilitated the prediction and design of new and more potent inhibitors. Altogether, our study confirmed that quinuclidinium carbamates are promising candidates for further development as CNS-active drugs, particularly for Alzheimer’s disease treatment.

## 1. Introduction

Central nervous system (CNS) diseases, particularly Alzheimer’s (AD) and Parkinson’s, are one of the great health-care challenges of the 21st century. According to the World Health Organisation (WHO), AD currently affects about 47 million people, and it is believed that this number will quadruple by 2050 [1,2]. AD is a multifactorial disease whose initiation and development is associated with many clinical features: deficits of the neurotransmitter acetylcholine (ACh), amyloid-β (Aβ) peptide deposits, oxidative stress, dyshomeostasis of biometals and hyperphosphorylated tau protein [3], and it is characterized by a continuous mental ability decline, behavioral dysfunction, failure to maintain daily living activities and, the most prominent feature, dementia [3,4,5]. Parkinson’s disease is a CNS disorder characterized by shaking, stiffness, and difficulty with walking, balance, and coordination, which is caused by a loss of dopaminergic neurons in certain parts of the brain [6].

The treatment of Alzheimer’s and Parkinson’s disease is based mainly on increasing the level of the neurotransmitter ACh by inhibiting enzymes that hydrolyze them, which is the most common approach in treatment [7,8,9,10]. There are two cholinesterases that can hydrolyze ACh: acetylcholinesterase (AChE) and butyrylcholinesterase (BChE) [11]. Both cholinesterases share the same catalytic mechanism of hydrolysis of choline esters, but their role in the organism is quite different. In nerve synapses, AChE terminates nerve impulse transmission by hydrolyzing ACh, while BChE does not have a clearly defined physiological function. The most likely function for BChE is that of a backup for AChE and protection of synaptic AChE from various kinds of xenobiotics [11,12,13]. These two enzymes share approximately 54% of their identity in the amino acid sequence [14,15], but substitutions of some amino acids in their active sites resulted in different catalytic activity, specificity for substrates, selectivity, and stereoselectivity of both AChE and BChE in their interaction with various esters [13,16,17].

**Figure 1:**
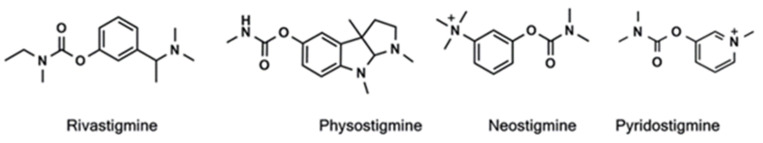
The structures of currently approved carbamate drugs for treatment of neurodegenerative disorders.

Due to their chemical and proteolytic stability, the ability to pass through cell membranes and form favourable intra- and intermolecular interactions in drug–target interaction, similarity in peptide bonds, or the ability to improve the biological activity of parent drugs, carbamates are used as a structural scaffold in many drugs approved by the US Food and Drug Administration (FDA) [18,19]. As esterases, cholinesterases interact with the esters of carbamic acids by a mechanism of action similar to ACh hydrolysis that takes place in three steps involving the formation of the Michaelis complex, the acylation of the enzyme, and its deacylation with water. Difference in action with carbamates lies in the rate of turnover of enzyme’s activity where the decarbamylation rate is much slower than deacetylation [20,21]. Today, there are four carbamate-based cholinesterase inhibitors (Figure 1) approved for the treatment of neurodegenerative diseases; two are active in the CNS (rivastigmine and physostigmine for the treatment of AD and Parkinson disease) and two are active in the peripheral nervous system (neostigmine and pyridostigmine for the treatment of Myasthenia gravis) [22].

However, current drugs for Alzheimer’s and Parkinson’s disease are single-target drugs that can achieve only temporary amelioration of symptoms instead of slowing or halting the disease progression and have shown various side effects depending on the drug used, and as such, they could not meet clinical needs [23,24]. In recent years, a strategy oriented to development of ligands that can interact with multiple disease-related targets has been proposed [25,26]. A very promising proposition is to use molecules that are AChE inhibitors (often using the structure of marketed AD drugs as a scaffold) with certain additional activities depending on fragments introduced into a molecule’s structure [24,25].

Quinuclidines are azabicyclic systems with a bridged nitrogen atom, and quinuclidin-3-ols and their esters can be considered bicyclic analogs of ACh, which explains their ability to act in cholinergic systems. Some quinuclidin-3-ol oximes were found to possess promising antidotal activity in poisoning by organophosphorus compounds; 3-oxoquinuclidinium derivatives [27] and bisquarternary pyridinium-containing quinuclidinium oximes [28] were shown to protect mice in poisoning by the organophosphorus compound soman. Recently, it was found that mono-oxime quinuclidinium-based compounds in combination with BChE have the potential to be used as bioscavengers of cyclosarin poisoning [29]. A protective effect against soman poisoning was determined for carbamate-based quinuclidine oximes 3-carbamyl-*N*-allylquinuclidinium bromide [30] and carbamoyl pyridinium ether [28]. Moreover, human AChE and BChE hydrolyze qunuclidinium acetates, benzoates, isonicotinate, and phthalates with the hydrolytic rates depending on its carboxy part of the molecules, where isoniconitate was the best AChE substrate [31,32]. Quaternized quinuclidine-3-ols [32] and their conjugates with imidazolium and pyridinium were determined to reversibly inhibit AChE [27,33]. Additionally, Cinchona-based alkaloids cinchonines and cinchonidines, quaternized with groups diverse in size, displayed anticholinesterase potency, pointing those compounds out as very potent human cholinesterase reversible inhibitors [34]. Two quinuclidinium-based carbamates were determined to be weak carbamylating agents of AChE [33].

**Figure 2:**
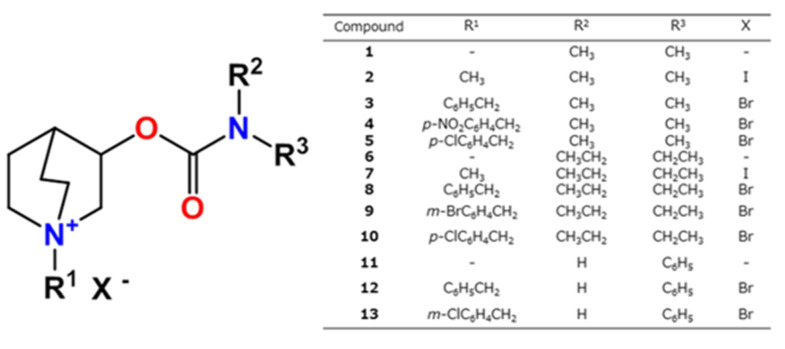
The structure of the synthesized compounds.

Due to the vital role of AChE inhibition in the treatment of AD and on its positive effect on cognitive symptoms, this study tested the inhibition potency of a series of new compounds with the aim to detect new structural scaffolds of AChE inhibitors to which additional pharmacophores may be added. As a continuation of our previous research, we synthesized thirteen compounds combining carbamate and quinuclidine moieties in one molecule and tested their inhibition potency toward human AChE and BChE (Figure 2). The obtained kinetic results were analyzed by multi-way analyses. To assess the ability of the tested compounds to be active in the CNS, their ability to cross the blood–brain barrier and their cytotoxicity on cells that represent the main models of individual organs were evaluated.

## 2. Materials and Methods

### 2.1. Chemicals

All chemicals, reagents, and solvents for the synthesis of quinuclidinium carbamates were purchased from commercial sources and used without further purification. Enzyme substrates acetylthiocholine iodide (ATCh) and propionylthiocholine iodide (PTCh) were purchased from Sigma-Aldrich, Steinheim, Germany, and thiole reagent 5,5′-dithiobis-2-nitrobenzoic acid (DTNB) from Sigma-Aldrich, St. Louis, MO, USA. All cell growth and cell culture supplements: RPMI-1640, Eagle’s Minimum Essential Medium (EMEM), Dulbecco’s Modified Eagle’s Medium/Nutrient Mixture F-12 Ham medium (DMEM F12), fetal bovine serum (FBS), penicillin/streptomycin (PenStrep), glutamine and non-essential amino acids (NEAA)) were purchased from Sigma-Aldrich, Steinheim, Germany.

#### 2.1.1. Enzyme Sources

Sources of AChE and BChE were native human erythrocytes and native human plasma, respectively. Blood from healthy individuals was collected and prepared as described previously [35]. Briefly, blood was collected in a heparinized tube and centrifuged at 2.5 rpm for 20 min at 4 °C to separate plasma from the erythrocytes. Erythrocytes were washed twice and diluted with phosphate buffer to the original blood volume. For measuring the AChE activity, erythrocytes hemolyzed by freezing were used.

#### 2.1.2. Cell Culture

All cell lines were obtained from the European Collection of Authenticated Cell Cultures cell-bank (ECACC). A549 cells (ECACC 86012804) were grown in RPMI-1640 medium supplemented with 10% (*v*/*v*) FBS, and 1% (*v*/*v*) PenStrep. HEK293 cells (ECACC 85120602) were grown in EMEM supplemented with 10% (*v*/*v*) FBS, 2 mM glutamine, 1% (*v*/*v*) PenStrep, and 1% (*v*/*v*) NEAA. SH-SY5Y cells (ECACC 94030304) were grown in DMEM F12 supplemented with 15% (*v*/*v*) FBS, 2 mM glutamine, 1% (*v*/*v*) PenStrep, and 1% (*v*/*v*) NEAA. All cells were grown at 37 °C in 5% CO_2_ atmosphere, the medium was changed every few days, and passages were done according to standard protocol [36]. Phosphate-buffered saline (PBS, pH 7.4) was prepared according to a standard recipe [37] and used for washing the cells when needed in the assays.

### 2.2. Synthesis of Compounds

Thin-layer chromatography was performed on aluminum oxide 60 F_254_ plates (Sigma-Aldrich, St. Louis, MO, USA) and visualized under UV light (254 nm) or by iodine fumes. Elemental analysis (CHN) was performed on a Perkin Elmer 2400 Series II CHNS analyzer (PerkinElmer, Inc., Waltham, MA, USA), and the purity of all compounds was ≥99%. Melting points were determined on a Melting Point B-540 apparatus (Büchi Labortechnik GmbH, Essen, Germany) and are uncorrected. FTIR (Fourier Transform Infrared Spectroscopy) spectra were recorded as KBr pellets on a Perkin-Elmer Spectrum Two (PerkinElmer, Inc., Waltham, MA, USA). NMR spectra were recorded on a Bruker Avance III HD 400 MHz/54 mm Ascend spectrometer (Bruker Corporation, Billerica, MA, USA) at 22 °C in CDCl_3_ and DMSO-*d*_6_. Chemical shifts are given in ppm downfield from tetramethylsilane as the internal standard. Coupling constants (*J*) are given in Hz. Splitting patterns are labeled as s (singlet), d (doublet), dd (doublet of doublets), t (triplet), q (quartet), or m (multiplet). Compounds 1, 6, and 11 were synthesized from quinuclidin-3-ol hydrochloride (≥98.0%, Fluka, Honeywell Research Chemicals, Charlotte, NC, USA) following published procedures [33,38], and the other compounds were prepared in a reaction of the appropriate carbamate with halides [39]. All halides were obtained from Sigma-Aldrich Co., St. Louis, MO, USA and used without further purification.

*N-Benzyl-3-(N,N-dimethylcarbamoyloxy)quinuclidinium bromide* (**3**) Yield: 78%; mp: 111.5–115.1 °C; IR (KBr) νν/cm^−1^: 3439; 3357; 3222; 2991; 2960; 2886; 1706; 1616; 1499; 1464; 1388; 1265; 1185; 1068; 984; 892; 770; 711; 711. ^1^H NMR (400 MHz, DMSO˗*d*_6_) *δ*/ppm: 1.79–2.13 (m, H5, H7); 2.23–2.29 (m, H4); 2.84 (s, CH3); 2.89 (s, CH3); 3.31–3.35 (m, H2; H6); 3.46–3.50 (m, H6, H8); 3.71–3.79 (m, H2); 4.53 (m, CH2bnz); 4.85–4.92 (m, H3); 7.49–7.56 (m, Hbnz). ^13^C NMR (DMSO˗*d*_6_) *δ*/ppm: 18.55 (C8); 20.89 (C5); 24.30 (C4); 36.06 (CH3); 36.48 (CH3); 52.51(C7); 54.29 (C6); 60.85(C2); 66.30 (C11); 68.08 (C3); 127.91 (C12); 129.48 (C15); 130.69 (C14, C16); 133.52 (C13, C17); 155.16 (C=O). CHN analysis/%: Anal. calcd. for (C_17_H_25_BrN_2_O_2_)/%: C 55.29; H 6.82; Br 21.64; N 7.59; O 8.66; Found: C 55.32; H 6.81; N 7.57.

*N-(4-Nitrobenzyl)-3-(N,N-dimethylcarbamoyloxy)quinuclidinium bromide* (**4**) Yield: 95%; mp: 200.5–204.7 °C; IR (KBr) ν/cm^−1^: 3107; 2962; 2886; 1706; 1607; 1524; 1491; 1392; 1349; 1272; 1188; 1096; 1048; 999; 940; 857; 767. ^1^H NMR (400 MHz, DMSO˗*d*_6_) *δ*/ppm: 1.82–2.09 (m, H5, H7); 2.24–2.30 (m, H4); 2.84 (s, CH3); 2.90 (s, CH3); 3.34–3.43 (m, H2); 3.45–3.62 (m, H6, H8); 3.75–3.82 (m, H2); 4.71 (q, CH2bnz); 4.84–4.90 (m, H3); 7.81–7.84 (m, H13, H17); 8.35–8.39 (m, H14, H16). ^13^C NMR (DMSO˗*d*_6_) *δ*/ppm: 18.51 (C8); 20.93 (C5); 24.68 (C4); 36.08 (CH3); 36.49 (CH3); 52.7 (C7); 54.6 (C6); 61.01 (C2); 68.02 (C11); 68.62 (C3); 124.33; 135.04;135.11; 149.04 (C12, C13,C14,C15,C16,C17); 155.14 (C=O). CHN analysis/%: Anal. calcd. for (C_17_H_24_BrN_3_O_4_)/%: C 49.28; H 5.84; Br 19.29; N 10.14; O 15.45; Found: C 49.20; H 5.86; N 10.15.

*N-(4-Chlorobenzyl)-3-(N,N-dimethylcarbamoyloxy)quinuclidinium bromide* (**5**) Yield: 75%; mp: 193.3–195.6 °C; IR (KBr) ν/cm^−1^: 3085; 2966; 2884; 1705; 1598; 1494; 1391; 1340; 1272; 1190; 1095; 1067; 999; 927; 898; 861; 766. ^1^H NMR (400 MHz, DMSO˗*d*_6_) *δ*/ppm: 1.75–2.14 (m, H5, H7); 2.21–2.32 (m, H4); 2.84 (s, CH3); 2.90 (s, CH3); 3.27–3.43 (m, H6, H2); 3.40–3.58 (m, H6, H8); 3.69–3.79 (m, H2); 4.53 (q, CH2bnz); 4.84–4.90 (m, H3); 7.49–7.66 (m, H13, H14, H16, H17). ^13^C NMR (DMSO˗*d*_6_) *δ*/ppm: 18.48 (C8); 20.9 (C5); 24.78 (C4); 36.06 (CH3); 36.48 (CH3); 52.43 (C7); 54.33 (C6); 60.82 (C2); 65.30 (C11); 68.07 (C3); 126.89; 129.53; 135.34; 135.71 (C13,C14,C17,C16); 155.144 (C=O). CHN analysis/%: Anal. calcd. for (C_17_H_24_BrClN_2_O_2_)/%: C 50.57; H 5.99; Br 19.79; Cl 8.78; N 6.94; O 7.93; Found: C 50.48; H 6.01; N 6.94.

*3-(N,N-diethylcarbamoyloxy)quinuclidine* (**6**) Yield: 64%; oil; IR (NaCl) ν/cm^−1^: 3676-3120; 2938; 2870; 1696; 1473; 1426; 1274; 1173; 1018; 979; 770. ^1^H NMR (400 MHz, DMSO˗*d*_6_) *δ*/ppm: 1.06 (s, CH3); 1.32–1.79 (m, H5, H7); 1.90–1.98 (m, H4); 2.55–2.82 (m, H6, H8; H2); 3.09–3.25 (m, CH2); 4.56–4.63 (m, H3). ^13^C NMR (DMSO˗*d*_6_) *δ*/ppm: 14.58; 13.93 (CH3); 19.72;24.17 (C5, C8); 25.56 (C4); 47.26 (C6, C7); 46.23 (CH2N); 55.76 (C2); 70.98 (C3); 155.144 (C=O).

*N-Methyl-3-(N,N-diethylcarbamoyloxy)quinuclidinium iodide* (**7**) Yield: 80%; mp: 142.1–144.7 °C; IR (KBr) ν/cm^−1^: 2966; 2881; 1698; 1531; 1476; 1428; 1379; 1275; 1170; 1105; 1081; 1008; 961; 766. ^1^H NMR (400 MHz, DMSO˗*d*_6_) *δ*/ppm: 0.97–1.18 (m, CH3); 1.78–2.08 (m, H5, H8); 2.25–2.35(m, H4); 2.87–3.05(m, NCH_3_); 3.15–3.31 (m, CH2, H7, H6); 3.44–3.5 (m, H2, H6); 3.78–3.86 (m, H2); 4.86–4.92 (m, H3). ^13^C NMR (DMSO˗*d*_6_) *δ*/ppm: 13.84 (CH3); 14.61 (CH3);18.65 (C8); 21.21 (C5); 24.07(C4); 41.47; 41.82 (CH2N); 51.21 (NCH3); 55.46; 56.09 (C7, C6); 62.76 (C2); 68.01(C3); 154.49 (C=O). CHN analysis/%: Anal. calcd. for (C_13_H_25_IN_2_O_2_)/%: C 42.40; H 6.84; I 34.46; N 7.61; O 8.69; Found: C 42.47; H 6.85; N 7.59.

*N-Benzyl-3-(N,N-diethylcarbamoyloxy)quinuclidinium bromide* (**8**) Yield: 86%; mp: 106.7–107.1 °C; IR (KBr) ν/cm^−1^: 3483; 3237; 2967; 2890; 1695; 1478; 1430; 1395; 1274; 1221; 1171; 1084; 1000; 940; 891; 767; 709. ^1^H NMR (400 MHz, DMSO˗*d*_6_) *δ*/ppm: 0.97–1.73 (m, Me); 1.76–2.14 (m, H5,H8); 2.22–2.36 (m, H4); 3.12–3.31 (m, H6, H7); 3.3–3.39 (m, CH2); 3.40–3.54 (m, CH2, H2); 3.73–3.82 (m, H2); 4.48–4.62 (m, CH2bnz); 4.87–4.96 (m, H3); 7.52 (s, CHbnz). ^13^C NMR (DMSO˗*d*_6_) *δ*/ppm: 14.59; 13.83 (CH3 Et); 20.91; 18.56 (C5, C8); 24.85 (C4); 41.45; 41.83 (CH2 Et); 54.27; 52.52 (C6, C7); 60.97 (C2); 66.28 (CH2bnz); 67.87 (C3); 127.93 (C1bnz);129.66; 130.68; 133.62 (CHbnz); 154.40 (C=O). CHN analysis/%: Anal. calcd. for (C_19_H_29_BrN_2_O_2_)/%: C 57.43; H 7.36; Br 20.11; N 7.05; O 8.05; Found: C 57.47; H 7.38; N 7.05.

*N*-(3*-Bromobenzyl)-3-(N,N-diethylcarbamoyloxy)quinuclidinium bromide* (**9**) Yield: 83%; mp: 144.3–147.5 °C; IR (KBr) ν/cm^−1^: 3429; 3370; 3243; 2974; 2882; 1684; 1631; 1569; 1438; 1393; 1277; 1216; 1168; 1076; 1002; 942; 891; 829; 797; 768; 717; 669; 617; 567; 447. ^1^H NMR (400 MHz, DMSO˗*d*_6_) *δ*/ppm: 0.96–1.17 (m, Me); 1.72–2.13 (m, H5, H7); 2.21–2.36 (m, H4); 3.14–3.4 (m, H6, H8); 3.3–3.39 (m, CH2); 3.40–3.58 (m, CH2, H2); 3.74–3.85 (m, H2); 4.46–4.62 (m, CH2bnz); 4.87–4.96 (m, H3); 7.45–7.79 (m, CHbnz). ^13^C NMR (DMSO˗*d*_6_) *δ*/ppm: 13.83; 14.61 (CH3 Et); 18.60; 20.91 (C5, C7); 24.85 (C4); 41.46; 41.84 (CH2 Et); 52.55; 54.51(C6, C8); 61.11 (C2); 65.26 (CH2bnz); 67.83 (C3); 122.53 (C14);130.51; 131.56; 132.68; 133.59; 135.94 (C15, 16, 17, 18, 19); 154.44 (C=O). CHN analysis/%: Anal. calcd. for (C_19_H_28_Br_2_N_2_O_2_)/%: C 47.92; H 5.93; Br 33.56; N 5.88; O 6.72; Found: C 47.96; H 5.93; N 5.89.

*N*-(4*-Chlorobenzyl)-3-(N,N-diethylcarbamoyloxy)quinuclidinium bromide* (**10**) Yield: 77%; mp: 92.6–94.1 °C; IR (KBr) ν/cm^−1^: 3421; 3368; 2970; 2932; 1698; 1475; 1430; 1393; 1317; 1277; 1225; 1171; 1091; 1020; 942; 890; 829; 767; 734; 590; 529; 487; 443. ^1^H NMR (400 MHz, DMSO˗*d*_6_) *δ*/ppm: 0.91–1.25 (m, Me); 1.69–2.13 (m, H5,H7); 2.19–2.39 (m, H4); 3.16–3.42 (m, H6, H8); 3.3–3.39 (m, CH2); 3.43–3.53 (m, CH2, H2); 3.72–3.85 (m, H2); 4.49–4.62 (m, CH2bnz); 4.87–4.94 (m, H3); 7.52–7.64 (m, CHbnz). ^13^C NMR (DMSO˗*d*_6_) *δ*/ppm: 13.83; 14.60 (CH3 Et); 18.59; 20.90 (C5, C7); 24.85 (C4); 41.46; 41.83 (CH2 Et); 52.38; 54.31(C6, C8); 60.95 (C2); 65.25 (CH2bnz); 67.84 (C3); 126.91 (C14);129.50; 135.36; 135.70 (Cbnz); 154.44 (C=O). CHN analysis/%: Anal. calcd. for (C_19_H_28_BrClN_2_O_2_)/%: C 52.85; H 6.54; Br 18.51; Cl 8.21; N 6.49; O 7.41; Found: C 52.84; H 6.53; N 6.48.

*N-Benzyl-3-(N-phenylcarbamoyloxy)quinuclidinium bromide* (**12**) Yield: 54%; mp: 65.3–67.9 °C; IR (KBr) ν/cm^−1^: 2887-3752; 1726; 1600; 1544; 1498; 1445; 1317; 1225; 1093; 1064; 895; 764; 706; 619; 509. ^1^H NMR (400 MHz, CDCl_3_) *δ*/ppm: 1.4–1.7 (m, H8); 1.85–1.87(m, H5); 1.9–2.1 (m, H4); 2.7–3.2 (m, H2, H7, H6); 3.2–3.4 (m, H2); 4.7–4.9 (m, H3); 7.0–7.1 (m, CHbnz); 7.2–7.5 (m, CHbnz). ^13^C NMR (CDCl_3_) *δ*/ppm:19.56 (C5); 24.63 (C8); 25.43 (C4); 47.44; 45.54 (C7, C6); 55.54 (C2); 72.18 (C3); 118.68; 123.45; 129.08; (CHbnz); 138.02 (Cbnz); 152.66 (C=O). CHN analysis/%: Anal. calcd. for (C_21_H_25_BrN_2_O_2_)/%: C 60.44; H 6.04; Br 19.15; N 6.71; O 7.67; Found: C 60.46; H 6.04; N 6.73.

*N*-(3*-Chlorobenzyl)-3-(N-phenylcarbamoyloxy)quinuclidinium bromide* (**13**) Yield: 76%; mp: 85.8–89.3 °C; IR (KBr) ν/cm^−1^: 3535-3261; 3183; 3049; 2966; 1727; 1600; 1544; 1491; 1445; 1317; 1225; 1092; 1064; 880; 832; 798; 748; 694; 567; 509; 436. ^1^H NMR (400 MHz, DMSO-*d*_6_): 1.8–2.0 (m, H8, H5); 2.1–2.2 (m, H5); 2.2–2.3 (m, H4); 3.2–3.6 (m, H2, H7, H6); 3.8–3.9 (m, H2); 4.4–4.6 (m, CH2bnz); 5.0–5.1 (m, H3); 7.0–7.8 (m, CHbnz); 9.9 (s, NH). ^13^C NMR (CDCl_3_) *δ*/ppm:18.42 (C5); 20.95 (C8); 24.71 (C4); 52.91; 54.46 (C7, C6); 60.79 (C2); 65.38 (CH2bnz); 67.76 (C3); 118.77; 123.25; 129.30; 130.75; 131.35; 132.36; 133.15 (CHbnz); 130.22; 133.98; (Cbnz); 153.1 (C=O). CHN analysis/%: Anal. calcd. for (C_22_H_27_BrN_2_O_2_)/%: C 61.26; H 6.31; Br 18.52; N 6.49; O 7.42; Found: C 61.39; H 6.32; N 6.48.

### 2.3. Cholinesterase Inhibition

#### 2.3.1. Enzyme Activity Measurement

Enzyme activities were determined using the Ellman spectrophotometric method [40] at 25 °C in 0.1 M phosphate buffer (pH = 7.4) with 0.3 mM DTNB as the thiol reagent. Substrate stock solutions, ATCh (10 mM), and PTCh (40 mM), and carbamates (50–100 mM), as well as their further dilutions, were prepared in water. Final concentrations of carbamates were in the range of 0.5–200 μM, while substrates were 1.0 mM and 4.0 mM for ATCh and PTCh, respectively. Final dilution of AChE and BChE was 500 and 300 times, respectably.

The extend of inhibition was determined by measuring the time dependence of cholinesterases inhibition by carbamates [16]. An inhibitor was added to the reaction mixture containing DTNB, buffer, and enzyme, and after a given incubation time, a substrate was added followed by measurement of the residual activity of the enzyme. Enzyme activity at “zero” time was measured after adding the enzyme to a reaction mixture containing DTNB, buffer, inhibitor, and substrate immediately before the start of the measurement. With the inhibited probes, the activities of the control probes, which did not contain an inhibitor, were measured.

The increase in absorbance was recorded at 436 nm for AChE or 412 nm for BChE [40,41], on a Cary 300 spectrophotometer (Varian, Inc., Mulgrave, Australia).

#### 2.3.2. Inhibition Constants Determination

**Scheme 1:**
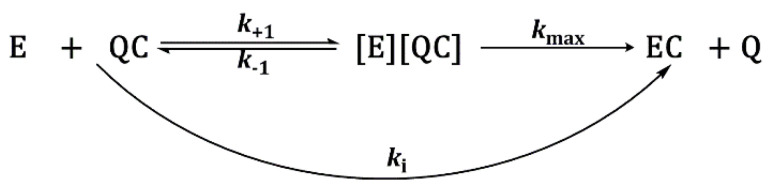
Progressive inhibition of cholinesterases by carbamates.

Enzyme inhibition proceeds according to the Scheme 1:

Where E, QC, [E] [QC], EC, and Q stands for free enzyme, inhibitor, Michaelis-type complex between enzyme and inhibitor, carbamylated enzyme, and leaving group, respectively. The first-order rate constants (*k*_obs_) were calculated by the linear regression analyses at any given inhibitor concentration ([QC]):

(1) $$ln\frac{v_{0}}{v_{i}}=k_{\mathrm{obs}}\cdot t$$

where *v*_0_ and *v*_i_ stand for the enzyme activity in the absence and in the presence of inhibitor at time *t*. When the *k*_obs_ was a linear function of [QC], the second-order inhibition rate constant (*k*_i_) was calculated from:

(2) $$k_{i}=\frac{k_{\mathrm{obs}}}{t}$$

when dependence of *k*_obs_ vs. [QC] was not linear, indicating the presence of a reversible enzyme–inhibitor complex, the maximum first-order inhibition rate constant (*k*_max_) and the dissociation constants of enzyme–inhibitor complex (*K*_a_) were determined from:

(3) $$k_{\mathrm{obs}}=\frac{k_{\max}\cdot \left[QC\right]}{K_{a}+\left[QC\right]]}$$

Then, the *k*_i_ constant was the ratio:

(4) $$k_{i}=\frac{k_{\max}}{K_{a}}$$

All kinetic parameters were calculated using the statistical package *GraphPadPrism 8* (Graph Pad Inc., San Diego, CA, USA).

### 2.4. Multivariate Analysis

Multivariate analyses of sampled molecular dynamics data were performed using the second-order tensor analysis tool principal component analysis (PCA). In PCA, the data matrix (or two-way array) ***X*** of rank *r* (usually not known) with mean centred columns that consists of *i* rows (compounds) and *j* variables (energy values) was decomposed as a sum of total of *r* matrices $t_{i}p_{i}^{\tau }$ with rank 1:

(5) $$X=\overset{r}{\underset{i=1}{\sum }}t_{i}p_{i}^{\tau }$$

where $t_{i}$ stands for score and $p_{i}^{\tau }$ stands for loading vectors. PCA finds the best linear projections for a high-dimensional set of data in the least squares sense. Scores represent projections of the original points on the principal component direction and can be used for classification, whereas loadings represent eigenvectors of data covariance (or correlation) matrix and can be used for the identification of variability among the data.

### 2.5. Machine Learning Procedure

Multivariate linear regression models using a linear combination of variables as well as the higher-order polynomial terms (up to the degree of 5) were constructed and validated. Matrices of coefficients B were calculated by singular value decomposition using the expression:

(6) $$B={\left(X^{\tau }X\right)}^{-1}X^{\tau }Y.$$

Each model was extensively tested by using the leave-one-out cross-validation procedure (LOO) and the coefficient of determination, standard error of regression, average *R*^2^ in LOO, and cross-validation mean squared error were computed. Based on these values, the most optimal representation model was selected.

### 2.6. In Silico Prediction of Blood–Brain Barrier (BBB) Penetration

The ability of synthesised quinuclidine carbamates to cross the blood–brain barrier (BBB) was estimated by calculating the molecular descriptors important for passive transport [42,43]: the logarithm of the octanol/water partition coefficient (logP), the molecular weight (MW), the polar surface area (PSA), the number of hydrogen bond donors (HBD), the number of hydrogen bond acceptors (HBA), and molecular flexibility characterized by the number of rotable bonds (RB). Parameters were determined in silico using the Chemicalize 2018 platform [44]. The obtained results were compared to the recommendations of physicochemical properties for successful central nervous system drugs [45].

### 2.7. Cytotoxicity of Carbamates

The cytotoxic profiles of tested carbamates were determined by measuring the succinate dehydrogenase mitochondrial activity of cells exposed to them [46]. We used the commercially available MTS detection reagent assay (CellTiter 96^®^ AQueous One Solution Cell Proliferation Assay, Promega, Madison, WI, USA). The procedure followed a previously described protocol [29]. All cell types were seeded at a density of 20,000 cells/well in 96-well plates one day before the experiment. On the day of the experiment, cells were exposed to the carbamates in a concentration range of 6.25–400 µM for 24 h. After a set time of incubation at 37 °C in a 5% CO_2_ atmosphere, cells were washed once with PBS buffer and 100 µL of corresponding medium, 20 µL of MTS reagent was added to each well, and after 3 h of incubation, the absorbance was read at 492 nm on an Infinite M200PRO plate reader (Tecan Austria GmbH, Salzburg, Austria). Data were evaluated from at least two separate experiments (each treatment in duplicate) by a nonlinear fit equation predefined in *GraphPadPrism 8* software (GraphPad Inc., San Diego, CA, USA). The results were expressed as a percentage of a cell death in carbamate-treated cells compared to the untreated cells.

## 3. Results and Discussion

### 3.1. Synthesis of Compounds

Thirteen quinuclidine carbamates differing in substituents at the quinuclidinium nitrogen atom and carbamoyl nitrogen atom were synthesized. We prepared a series of disubstituted (*N*,*N*-dimethyl, compounds **1**–**5** and *N*,*N*-diethyl, compounds **6**–**10**) and monosubstituted carbamates (*N*-phenyl, compounds **11**–**13**), as shown in Figure 2. The syntheses of quinuclidinium carbamates started from the commercially available quinuclidine-3-ol. Carbamates were prepared by the reaction of alcohol and differently substituted carbamoyl chlorides [33] or isocyanate [38]. To prepare quaternary compounds, a Menshutkin reaction was employed. Compounds **1**, **6**, and **11** were converted to quaternary ammonium salts by a reaction with the appropriate alkyl/aryl halide [39]. The best conditions were obtained in the reaction of carbamates with 1 eq. of appropriate alkyl/aryl halide in dry tetrahydrofurane at reflux temperature and under a nitrogen atmosphere. The products were obtained in 54–95% yield as solids that precipitated from the reaction mixture using diethyl ether. Structures of prepared compounds were deduced from IR, one-, and two-dimensional NMR spectra. Compounds **3**–**10**, **12**, and **13** are new compounds and have not been previously described in the literature.

### 3.2. Inhibition of Cholinesterases

**Figure 3:**
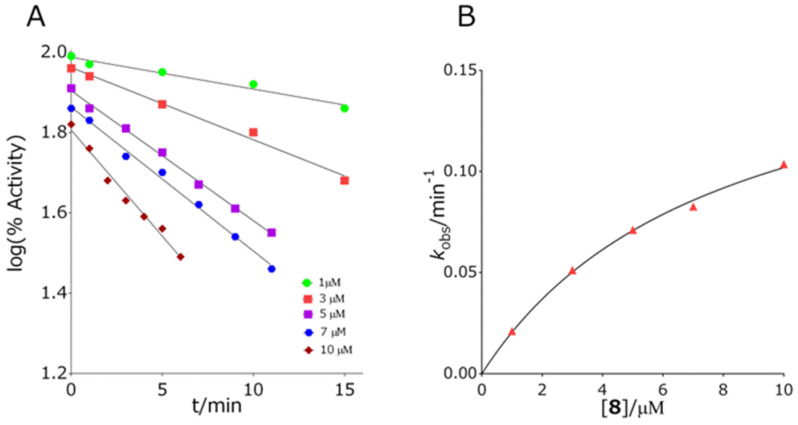
Representative experiment of the inhibition of butyrylcholinesterase (BChE) by compound **8**. Panel (**A**): points indicate the logarithm of residual activity, while the slope of the lines determines the first-order rate constant *k*_obs_. Panel (**B**): The constants *k*_obs_ were plotted as a function of carbamate concentration wherefrom the second-order rate constant, *k*_i_, was calculated.

All of the tested quinuclidine carbamates displayed a time-dependent inhibition of both cholinesterases, AChE and BChE. Inhibition followed first-order kinetics at any given inhibitor concentration (Figure 3 panel A).

For AChE, eleven out of thirteen quinuclidine carbamates displayed a nonlinear dependence of the first-order rate constant (*k*_obs_) on carbamate concentration (Figure 3 panel B) allowing for the determination of the maximal first-order rate constant of carbamylation, *k*_max_, and dissociation constant of the enzyme–carbamate Michaelis type of complex, *K*_a_ (Table 1). For compounds **8** and **11**, *k*_obs_ was a linear function of carbamate concentration. The overall inhibition rate constant *k*_i_, which represents the first step in carbamates hydrolysis, i.e., the carbamylation rate constant, expresses the measure of inhibition potency of quinuclidinium carbamates. The *k*_i_ constants for the tested quinuclidinium carbamates and AChE were in range of (1.0–15) 10^3^ M^−1^ min^−1^, (median 3.5·10^3^ M^−1^ min^−1^). The most potent AChE inhibitor was diethyl-carbamate quaternized with a benzyl group, compound **8**, while the least potent was the non-quaternized phenyl carbamate **11**, being 15 times less potent than **8**. Interestingly, for both carbamates, the first-order rate constant, *k*_[QC]_, displayed linear dependence on the carbamate concentration pointing to the fact that the concentration of the reversible enzyme–inhibitor complex during inhibition is negligible. Generally, the derived *k*_i_ constants corresponded very well to that determined for rivastigmine, the carbamate currently in use in AD treatment, having constants in range of 1.13–4.54 10^3^ M^−1^ min^−1^ [47,48,49,50,51]. However, compared to the inhibition potency of physostigmine, in use for Parkinson’s disease treatment, quinuclidinium carbamates are about three orders of magnitude less potent AChE inhibitors (Table 2) [52]. Although the *k*_i_ constants of all of the tested carbamates did not differ much, a certain trend was noticed. If the carbamates are grouped with respect to the amine part of the carbamate group, it can be observed that the quaternization of the quinuclidinium nitrogen leads to a slight increase of the overall rate of carbamylation. This observation is most evident by carbamates with a phenyl group in the amino part of the molecule where quaternization of quinuclidinium nitrogen increased the carbamylation rate by about 3.5 times. It is known that the rate of cholinesterase carbamylation is determined by the entrance of the carbamate and its orientation into the active site of the enzyme [53]. It seems that these two activities in the case of tested quinuclidinium carbamates were dictated primarily by the quinuclidine part of the carbamates. Although the overall inhibition rate constants of AChE were very similar, the intrinsic carbamylation constants differed; AChE had the highest *k*_max_ and *K*_a_ for compound **3**, but consequently, its corresponding *k*_i_ was close to the median.

Inhibition of BChE displayed a nonlinear dependence of the first-order rate constant and carbamates concentration for all tested quinuclidine carbamates, with *k*_i_ constants in the range (1.5–24) × 10^3^ M^−1^ min^−1^ (Table 1). An exception was compound **7**, where *k*_obs_ was a linear function of the carbamate concentration. The most potent inhibitor was compound **8**, which was an BChE inhibitor that was about 6 times more potent than the other tested carbamates.

The ratio of overall inhibition rate constants for BChE and AChE describes the inhibition selectivity of the tested carbamates (Table 1). Generally, the tested quinuclidinium carbamates did not displayed pronounced selectivity either of the cholinesterase; the ratio of the overall inhibition rate constants ranged from 0.42 to 1.8. Only in the case of compound **11** was a three times higher preference to BChE determined, which can be attributed to the low inhibition potency to AChE. This is not a downfall or disadvantage, since currently used cholinesterase inhibitor physostigmine is also non-selective.

### 3.3. Multivariate Analysis and Activity Models

A relationship between the structure of compounds and the bioactivity is crucial for understanding the mechanism of action and establishing prediction models. Dissociation constants of enzyme–inhibitor complex *K*_a_ were used as dependent variables for the estimation of quinuclidine carbamates activities. These constants were regressed on the theoretically computed energy fingerprints of all compounds whose principal components were calculated by the second-order tensor decomposition tool PCA. Energies collected during molecular dynamics trajectories were arranged in a data matrix, and the PCA on the covariance matrix was carried out using our multivariate analysis code [54] based on the NIPALS algorithm [55].

**Figure 4:**
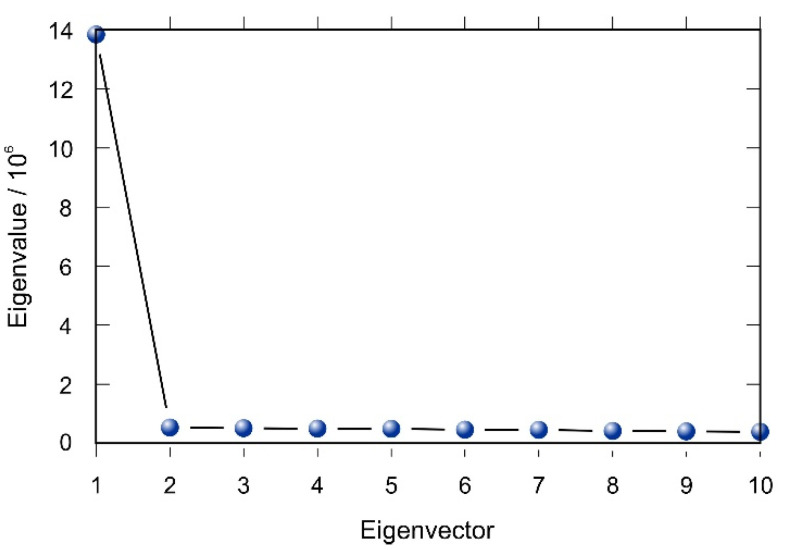
Screen plot for principal component analysis PCA of the compound energy fingerprints collected during molecular dynamics simulations.

The first two principal components of compound energies described more than 75% of the total variance (Table 2). Inspection of the scree plot revealed that these two principal components will be sufficient as independent variables for multivariate regression (Figure 4).

**Figure 5:**
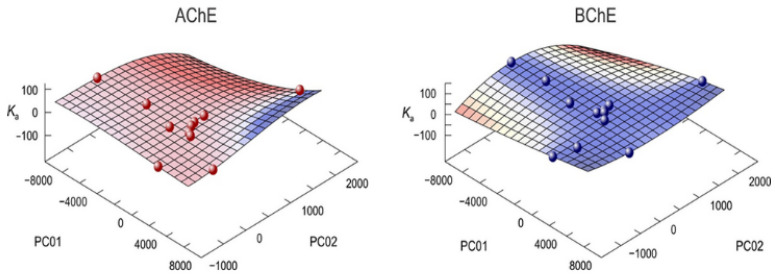
Multivariate regression models of dissociation constant of compounds for acetylcholinesterase (AChE) and butyrylcholinesterase (BChE).

An extensive machine learning procedure was applied for various multivariate linear regression models (up to polynomial degree of 5). Each of these models was validated by leave-one-out cross-validation technique (LOO). For every computed model, coefficient of determination and standard error of regression as well as the mean squared errors of prediction were computed, and the optimal model was selected. The mean squared errors of prediction determined by the LOO method were the lowest in the case of the third-order polynomial models. These models for AChE and BChE are presented in Figure 5.

LOO was bigger than for the model itself ensuring that there is no overfitting. The equivalent model for BChE described 90% of the variations in the data. The average *R*^2^ in the latter case was 92%, verifying again the validity of the model. These models can be used for predicting the bioactivity of new compounds and for the design of better inhibitors.

### 3.4. The BBB Penetration Ability of Tested Quinuclidinium Carbamates

**Figure 6:**
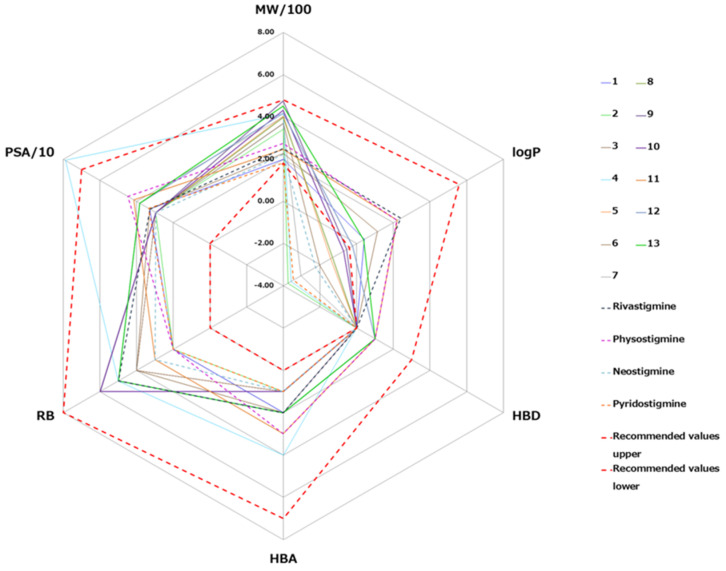
Radar plot of physicochemical properties (molecular weight, MW; lipophilicity coefficient, logP; number of hydrogen bonds donors, HBD, and acceptors HBA; rotatable bonds, RB; polar surface area, PSA) of the tested carbamates. The recommended values for the CNS-active drugs are presented by a dashed red line [56,57].

The ability of quinuclidinium carbamates to cross the BBB was assessed by comparing the calculated values of six physicochemical descriptors of compounds with the recommended values obtained for known CNS-active drugs [41]. CNS-active drugs generally have a molecular weight lower than 450 g moL^−1^, moderate hydrophobicity (logP < 5), less than three hydrogen bonds donors (HBS) and less than seven hydrogen bond acceptors (HBA), less than eight rotatable bonds (RB) and are less polar (polar surface area (PSA) < 70 Å^2^) than drugs that are not active in the CNS. A comparison of the obtained values for molecular descriptors for the tested carbamates and recommended values [45,56,57], for known CNS-active drugs, is shown at a radar plot in Figure 6.

Values of all molecular descriptors of tested carbamates were favorable and in the range of the upper recommended values of “Rule of 5” molecular descriptors values [56]. The only exception was the polar surface area of compound **4** that was outside of the recommended range. Regarding the lower recommended values for molecular descriptors, five (**1**, **6**, **11**, **12** and **13**) were favorable for all quinuclidinium carbamates. The hydrophobicity of carbamates **2**, **3**, **4**, **5**, **7**, **8**, **9** and **10** was below the lower recommended value, which reduces the possibility of those carbamates to cross the BBB. The values of the tested carbamates were compared to those calculated for carbamates currently in use for the treatment of neurodegenerative diseases: rivastigmine, physostigmine, pyridostigmine, and neostigmine (Figure 6). For five carbamates, compounds **1**, **6**, **11**, **12**, and **13**, the molecular descriptors were within the recommended range, close to that of CNS-active drugs rivastigmine and physostigmine. Compounds **1**, **6**, and **11** are the only non-quaternized compounds; i.e., they do not have a permanent positive charge known to prevent passive diffusion through the BBB. Compounds **12** and **13** do have a permanent charge, but it seems that it is counterbalanced by their additional aromatic rings. The hydrophobicity of the rest of the quinuclidinium compounds was close to that determined for pyridostigmine and neostigmine, the carbamates that target cholinesterases in the peripheral nervous system.

### 3.5. Cytotoxicity

**Figure 7:**
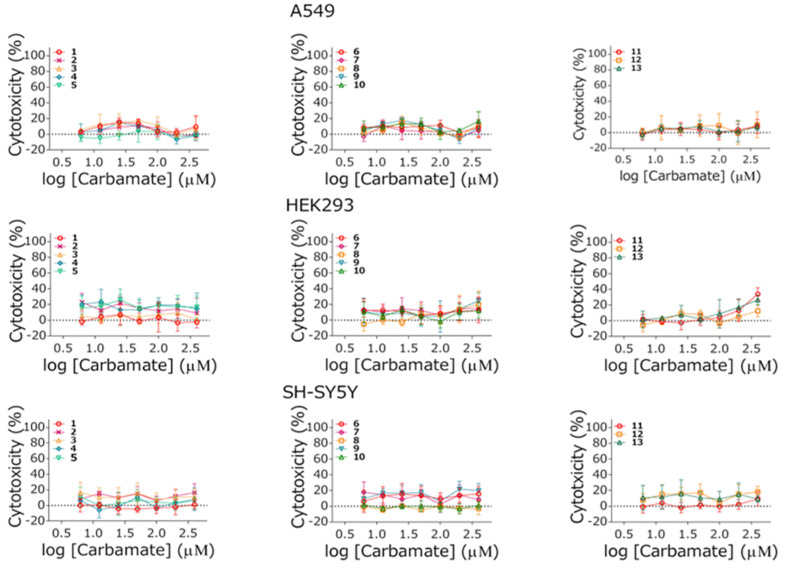
Cytotoxicity of tested carbamates in concentrations 6.25–400 μM on A549, HEK293 and SH-SY5Y cells. Data are given as a mean ± SE (*n* = 6) of two independent experiments.

The cytotoxic effect of quinuclidinium carbamates was evaluated on A549, HEK293, and SH-SY5Y in a 24 h exposure period. The concentration range was selected to correspond to the one used in vitro kinetic experiments of their cholinesterase inhibition potency testing. The obtained results are given in Figure 7. As the results indicate, none of the tested compounds induced a significant cytotoxic effect on either of the cell lines in the concentration range tested (up to 400 µM).

## 4. Conclusions

A series of thirteen quinuclidine-based carbamates, ten of which were new compounds, were synthesized and evaluated as potential CNS active substances. All of the synthesized compounds proved to be potent dual inhibitors of both AChE and BChE displaying a time-dependent inhibition of both cholinesterases and demonstrating the formation of a covalent bond with the active site serine. In silico prediction of BBB passive transport has showed that non-quaternized and quaternized phenyl-carbamates could cross the BBB and be therefore CNS active. Furthermore, cytotoxicity assays showed that all of the tested compounds were non-toxic on all cell lines. Based on our results, it can be concluded that non-quaternized dimethyl-, diethyl-, and phenyl quinuclidine carbamates, as well as quaternized phenyl carbamates can be considered potential candidates for further evaluation as potential cholinesterase-based drugs, or as a good starting point in the design and synthesis in terms of developing new CNS-active drugs with improved inhibition potency toward cholinesterases. The quaternized dimethyl- and diethyl- quinuclidine carbamates, regarding their dual inhibition potency and non-cytotoxicity, could be candidates for targeting cholinesterases in the peripheral nervous system. Based on these data, the regression models for the prediction of bioactivity were established and validated. In both cases, the average *R*^2^ in the LOO procedure was higher than 90%, as well as these values were higher than for the model itself, ensuring that there was no overfitting. An equivalent model for BChE described 90% of the variations in the data. The average *R*^2^ in the latter case was 92%, again verifying the validity of the model. These models present a sound tool that can be used for the prediction and design of new and better inhibitors.

## Figures and Tables

**Table 1:** Inhibition of acetylcholinesterase (AChE) and butyrylcholinesterase (BChE) by quinuclidinium carbamates **1**–**13**.

Compound	AChE			BChE			*k_i(BChE)_*/*k_i(AChE_*
	*k*_i_∙10^3^ [M^−1^min^−1^]	*K*_a_ [μM]	*k*_max_ [min^−1^]	*k*_i_∙10^3^ [M^−1^min^−1^]	*K*_a_ [μM]	*k*_max_ [min^−1^]	
**1**	2.6 ± 0.6	72 ± 15	0.19 ± 0.02	3.1 ± 0.7	60 ± 9	0.20 ± 0.02	1.3
**2**	5.5 ± 1.4	22 ± 5	0.12 ± 0.01	5.4 ± 1.5	18 ± 4	0.095 ± 0.007	0.98
**3**	3.4 ± 1.3	124 ± 63	0.34 ± 0.11	4.9 ± 0.7	32 ± 4	0.16 ± 0.01	1.4
**4**	5.9 ± 2.5	25 ± 10	0.15 ± 0.03	7.5 ± 1.9	15 ± 4	0.13 ± 0.01	1.3
**5**	5.5 ± 1.4	29 ± 7	0.16 ± 0.01	3.6 ± 0.8	80 ± 17	0.29 ± 0.03	0.65
**6**	2.9 ± 0.9	76 ± 19	0.22 ± 0.03	3.8 ± 0.9	41 ± 9	0.16 ± 0.01	1.3
**7**	3.5 ± 1.1	46 ± 13	0.16 ± 0.02	1.5 ± 0.0	-	-	0.42
**8**	15 ± 1	-	-	24 ± 6	7.9 ± 1.9	0.19 ± 0.02	1.6
**9**	6.8 ± 1.5	40 ± 8	0.27 ± 0.03	7.3 ± 2.1	32 ± 9	0.23 ± 0.03	1.0
**10**	3.2 ± 0.7	61 ± 13	0.20 ± 0.02	3.4 ± 2.0	46 ± 4	0.16 ± 0.05	1.0
**11**	1.0 ± 0.2	-	-	3.0 ± 0.9	65 ± 18	0.20 ± 0.03	3.0
**12**	3.7 ± 0.8	25 ± 5	0.09 ± 0.01	3.1 ± 1.1	103 ± 31	0.32 ± 0.05	0.83
**13**	3.2 ± 0.5	88 ± 13	0.28 ± 0.02	3.1 ± 1.0	89 ± 22	0.28 ± 0.04	0.98
Rivastigmine [48]	4.54			333			73
Physostigmine	4900 ± 380	66 ± 27	0.32 ± 0.22	2800 ± 940	250 ± 24	0.61 ± 0.22	1.8

**Table 2:** Total variance represented by principal components calculated for a set of compound energies from the molecular dynamics simulations.

Principal Component	Variance/%	Total/%
PC01	72.28	72.28
PC02	2.79	75.07
PC03	2.66	77.74
PC04	2.60	80.34
PC05	2.56	82.90

## Data Availability

Data sharing is not applicable to this article.
